# Green Coffee Bean Extract Normalize Obesity-Induced Alterations of Metabolic Parameters in Rats by Upregulating Adiponectin and GLUT4 Levels and Reducing RBP-4 and HOMA-IR

**DOI:** 10.3390/life12050693

**Published:** 2022-05-06

**Authors:** Esraa M. Seliem, Mohamed E. Azab, Randa S. Ismail, Abeer A. Nafeaa, Badriyah S. Alotaibi, Walaa A. Negm

**Affiliations:** 1 Department of Physiology, Faculty of Veterinary Medicine, Benha University, Benha 13512, Egypt; israa.magdy18@fvtm.bu.edu.eg (E.M.S.); mohamed.azab@fvtm.bu.edu.eg (M.E.A.); randa.ahmed@fvtm.bu.edu.eg (R.S.I.); abir.nafe@fvtm.bu.edu.eg (A.A.N.); 2 Department of Pharmaceutical Sciences, College of Pharmacy, Princess Nourah Bint Abdulrahman University, P.O. Box 84428, Riyadh 11671, Saudi Arabia; 3 Department of Pharmacognosy, Faculty of Pharmacy, Tanta University, Tanta 31111, Egypt

**Keywords:** adiponectin, adipose tissue, GLUT4, *Coffea arabica*, insulin resistance, inflammation, retinol-binding protein

## Abstract

Obesity is a serious public health issue worldwide. Finding safe and efficacious products to reverse obesity has proven to be a difficult challenge. This study showed the effects of *Coffea arabica* or green coffee bean extract (GCBE) on obesity disorders and the improvement of obesity-induced insulin resistance, dyslipidemia, and inflammation. The active constituents of GCBE were identified via high-performance liquid chromatography. Twenty-four male albino Wistar rats were divided into two groups. The first group (Group I) was fed a control diet, whereas the second group was fed a high-fat diet (HFD) for eight weeks till obesity induction. The second group was equally subdivided into Group II, which received HFD, and Group III, which received HFD + GCBE for another eight weeks. The body and organ weights of the animals were measured, and blood and adipose tissue samples were collected for analysis. The results indicated that the administration of GCBE significantly decreased the body and organ weights. Furthermore, it had an ameliorative effect on serum biochemical parameters. It dramatically reduced total cholesterol, triacylglycerol, low-density lipoprotein cholesterol, very low-density lipoprotein cholesterol, glucose, and insulin levels. In addition, an improvement in homeostasis model assessment-insulin resistance and an enhancement of high-density lipoprotein cholesterol levels were observed compared with the HFD group. In addition, the group treated with GCBE exhibited a marked increase in serum levels of adiponectin (an anti-inflammatory adipokine). In addition, a considerable reduction in adipocyte hypertrophy was found following GCBE treatment. Remarkably, the administration of GCBE resulted in a remarkable decrease in the expression of RBP4 (a pro-inflammatory cytokine), whereas an increase in GLLUT4 expression was observed in the adipose tissue. This improved insulin resistance in GCBE-administered HFD rats compared with other HFD rats. Our study showed that GCBE exhibits anti-obesity activity and may be used as a natural supplement to prevent and treat obesity and its associated disorders.

## 1. Introduction

Obesity is a severe health concern accompanied by immeasurable social costs. The incidence of obesity and its comorbidities has increased globally at an alarming rate. Obesity and being overweight are the world’s fifth top cause of death. However, it is becoming a significant threat that has a tremendous impact on the economy and quality of life [1]. It is a complicated condition aggravated by genetic, environmental, lifestyle, and nutritional factors that promote a chronic positive energy balance and expansion of body fat mass. Obesity contributes to metabolic disorders, including diabetes, hypertension, and heart disease. In addition, chronic conditions such as osteoarthritis, sleep apnea, stroke, certain malignancies, and inflammation-based pathologies may occur [2]. Abnormal or excessive buildup of fat in the adipocytes due to their hypertrophy and hyperplasia [3] can result from a high-calorie diet accompanied by a low expenditure of energy [4].

Adipose tissue is a crucial endocrine organ with active metabolism that has recently been recognized as a vital energy storage organ. Its endocrine function is important to maintain energy balance and homeostasis by producing pro and anti-inflammatory adipokines by adipocytes that play a vital role. Systemic inflammation, insulin resistance, and obesity-related metabolic diseases emerge when pro-inflammatory adipokines are produced and secreted in abundance [3].

Obesity is the leading risk factor for insulin resistance [5]. It is associated with low-grade and chronic inflammation, a physiological condition characterized by high concentrations of pro-inflammatory cytokines (e.g., RBP4, leptin, and IL-6) in tissues or the circulation and linked to insulin resistance [6]. The major contributors to the inflammatory process occurring in the adipose tissues are immune cells, particularly macrophages [7].

Obesity-related inflammation begins with an increase in macrophage infiltration into the adipose tissues and polarization, which includes modification of the macrophages of adipose tissue (M2). Unlike pro-inflammatory macrophages (M1), which maintain an inflammatory environment by secreting numerous pro-inflammatory mediators (e.g., RBP4, leptin, and IL-6), M2 macrophages perform anti-inflammatory functions (e.g., release adiponectin and IL-10) [8].

Because obesity increases clinical and economic burden, obesity management is becoming extremely important. Conventional therapy for obesity that provokes insulin resistance and inflammation primarily involves chemical drugs and surgical intervention, with numerous side effects and a high risk of recurrence [1]. Thus, evaluating the therapeutic value of natural products in treating and preventing obesity focuses on our research and identifying compounds with negligible side effects [9,10]. Consuming foods rich in bioactive anti-inflammatory components, such as polyphenols, reduces inflammation. Indeed, multiple cellular, animal, and human studies have provided evidence that dietary bioactive substances promote thermogenesis and energy expenditure by acting as antioxidants and anti-inflammatory agents. They also reduce oxidative stress and inflammation, which further enhances the weight loss effect and/or leads to the reduction of metabolic disorders [3]. Furthermore, the ability of polyphenols to interact directly or indirectly with adipose cells may explain the anti-obesity effects of polyphenol-rich diets [3].

Coffee is the most popular drink in the world, and it contains a variety of phytochemicals. Coffee consumption was shown to be beneficial for a variety of health problems, including metabolic syndrome (Mets), vascular function, and type 2 diabetes (T2DM) [11]. Green coffee, which refers to unroasted coffee beans [12], has been suggested to have the ability to prevent Mets [13] and T2DM [14]. Furthermore, several studies have demonstrated an alleviating effect of green coffee on some Mets components, including blood pressure [15], blood glucose [16,17], and lipid profile [16,17,18], as well as the leading etiological causes of Mets, such as insulin resistance and obesity [16].

Phenolic compounds, particularly chlorogenic acids (CGA), are phytochemicals abundantly found in coffee [11]. Green coffee bean extract (GCBE) is isolated from unroasted green coffee beans, resulting from a significant proportion of CGA depletion during the roasting process [19]. GCBE stimulates weight loss, promotes liver cells to accelerate fat metabolism, and reduces fat accumulation in the body without the requirement of calorie restriction. The general health benefits of GCBE are attributed to its distinctive polyphenols [12]. Animal studies have shown that CGA exhibits anti-obesity, antidiabetes [20], and antilipidemic characteristics [21,22] and ameliorates the effects of insulin resistance [23]. Furthermore, CGA has been shown to lower blood pressure [24] and postprandial glucose absorption in humans [25]. It is also believed that GCBE may alter adipokine levels [26]. In this study, the identification and quantitation of some phenolic compounds of GCBE were performed using high-performance liquid chromatography (HPLC)–diode-array detection (DAD). We assessed the anti-obesity properties of GCBE in rats fed with a high-fat diet (HFD). Improvements in the lipid profile and adipose tissue morphology were also evaluated. Finally, the effect of CGBE on insulin resistance and inflammation-related genes was determined.

## 2. Materials and Methods

### 2.1. GCBE Preparation

Green coffee bean or *Coffea arabica* was purchased from Shahin Coffee Markets, Egypt, and was confirmed by Dr. Esraa Ammar, Plant Ecology lecturer, Faculty of Science, Tanta University. A voucher sample (No. GP-A2021-018) was kept in the herbarium of the Pharmacognosy Department. Green coffee beans were ground to obtain a fine powder. The powder was extracted using the hot infusion method to extract *C. arabica* phenolic compounds. One Kg of powder was extracted with 5 L of hot distilled water (80 °C) in a water bath with shaking for 15 min, filtered with Whatman filter paper 1 (Sigma-Aldrich, St. Louis, MO, USA) lyophilized to obtain a powdered residue of GCBE. The yield (*w*/*w*) of aqueous extract was 34.88%. The residue was kept in a −20 °C freezer for further in-vivo investigation.

### 2.2. Drugs and Chemicals

All the chemicals and solvents used in this work were bought from Sigma-Aldrich and were of high analytical quality (St. Louis, MO, USA). The standard phenolic acids used were obtained from Sigma-Aldrich, USA.

### 2.3. HPLC-DAD of Coffea Arabica Extract

The Agilent Technologies 1100 series liquid chromatography includes an autosampler and a diode-array detector. Eclipse XDB-C18 was used as the analytical column (150 4.6 µm; 5 µm) with a C18 guard column (Phenomenex, Torrance, CA, USA). Acetonitrile (solvent A) and 2% acetic acid in water (solvent B) made up the mobile phase. The flow rate was held constant at 0.8 mL/min for a total run of 70 min, and the gradient programmer was as follows: 100% B to 85% B in 30 min, 85% B to 50% B in 20 min, 50% B to 0% B in 5 min, and 0% B to 100% B in 5 min. The injection volume was 50 µL, and peaks for benzoic acid and cinnamic acid derivatives were found simultaneously at 280 and 320 nm. Before injection, all samples were filtered using a 0.45 µm Acrodisc syringe filter (Gelman Laboratory, MI, USA). Congruent retention lengths and UV spectra were used to identify the peaks, then compared to the standards.

### 2.4. Experimental Animals and Ethical Approval

Twenty-four male albino Wistar rats (weighing 160–180 g) were used in this experiment. Rats were provided from the Laboratory Animals Research Center of the Center of Excellence for Scientific Research, Faculty of Veterinary Medicine, Benha University, Egypt. Separate stainless-steel cages were used to house the animals. Food and water were provided ad libitum. The animals were housed in a climate-controlled setting with a 12-h light/dark cycle (room temperature of 25 °C ± 2 °C, humidity maintained at 55% ± 5%).

The animals were provided one week to acclimate before initiating the experiments. During this period, all the animals were fed a balanced diet. Experiments on the animals were carried out with utmost care to avoid causing any pain or distress to the animals. The investigation was carried out following the criteria for the care and use of laboratory animals, which were authorized by the Research Ethical Committee (Faculty of Veterinary Medicine, Benha University, Approval Code No. BUFVTM 02-09-21).

### 2.5. Experimental Design

Following a period of acclimatization, the rats were either fed a control diet (CD; n = 8, AIN-76A diet #100000) or HFD (n = 16, rodent diet with 40% beef tallow modified AIN-76A diet #101556) for eight weeks to induce obesity [27]. Throughout the experiment, two fresh diets were prepared regularly. For HFD, the powdered chow was combined with fat until it was homogenous and had a dough-like consistency. A paste injector was used to shape the dough, and the resulting chow blocks were dried and fed to the animals [28].

The rats were separated after obesity induction into three groups (8 rats each). The following three groups were evaluated over eight weeks: Group I (control group) rats were given a control diet. Group II (HFD group) rats received HFD and distilled water orally daily by the same dosage regimen as the GCBE group. Group III (HFD + GCBE group) rats received HFD and 200 mg of GCBE /kg BW orally [16]. The rats were inspected every day, and the bodyweight of overnight fasting animals was registered weekly from the onset to the completion of the experiment.

### 2.6. Sampling

After eight weeks of GCBE administration, the rats were obliged to fast for the entire night. Next, they were anesthetized and sacrificed by collecting blood from the heart (intracardiac blood). Blood was drawn into serum separator and clot activator vials for lipid profiling and hormone level measurements. The serum was maintained at −20 °C until analysis after centrifugation at 4000 rpm for 15 min. Serum glucose levels were immediately measured after serum separation. Furthermore, the heart, liver, kidney, and spleen were immediately harvested and weighed. In addition, a portion of fresh adipose tissue was dissected and immediately preserved in 10% neutral formalin for histopathological investigation, whereas another portion was immediately frozen at −80 °C for real-time PCR analysis.

### 2.7. Serum Metabolite Study

Serum biochemical indicators of metabolic disorders associated with obesity were assessed. Total cholesterol (TC) level according to the method described by Allain et al. [29], triacylglycerol (TAG) level according to the method described by Fossati and Prencipe [30], and high-density lipoprotein cholesterol (HDL-C) level following the procedure of Lopes-Virella et al. [31]. Low-density lipoprotein cholesterol (LDL-C) and very-low-density lipoprotein cholesterol (VLDL-C) were evaluated by the method of Friedewald et al. [32]. Fasting serum glucose (FSG) level was assessed according to the procedure reported by Trinder [33] using an enzymatic colorimetric method and diagnostic kits (Bio-diagnostic company, Cairo, Egypt) according to the manufacturer’s instructions. Serum insulin levels were evaluated by a sandwich enzyme-linked immunosorbent assay using rat insulin ELISA kits (Abnova Corporation, Taipei, Taiwan).

Insulin resistance was estimated using mathematical models based on fasting insulin (µU/mL) and fasting glucose (mmol/L) levels. The following equation determined the homeostasis model assessment for insulin resistance (HOMA-IR) [34]:

insulin concentration × glucose concentration/22.5

Serum adiponectin value was computed quantitatively using the rat adiponectin ELISA kit (BioVision, Milpitas, CA, USA).

### 2.8. Adipose Tissue Histopathology

After resecting adipose tissues, 10% neutral-buffered formalin was used to fix the specimens. The fixed adipose tissue was immersed in paraffin. The resulting tissue blocks were sliced to a 4-µm thickness, and then hematoxylin and eosin were used to stain the tissue (H&E), followed by conventional histopathological procedures for light microscopic inspection. The sections were imaged under 100× and 400× magnifications.

### 2.9. Extraction of Total RNA and Quantitative Real-Time PCR

The Gene JET RNA Purification Kit (Thermo Scientific, Waltham, USA) was used to extract total RNA from the adipose tissues according to the manufacturer’s instructions. The isolated RNA was reverse transcribed into cDNA using the TOPscriptTM cDNA Synthesis Kit (enzynomics, Daejeon, Korea). The expression of RBP4 and GLUT4 was measured using a QIAGEN Rotor-Gene Q 5plex system (QIAGEN, Hilden, Germany) with TOPrealTM qPCR 2X PreMIX (SYBR Green with low ROX) (enzynomics, Daejeon, Korea) according to the manufacturer’s guidelines. Primers were purchased from Willowfort (Birmingham, UK), and the sequences are presented in Table S1.

The cycling conditions for real-time PCR included an activation step at 95 °C for 15 min followed by 45 cycles of 95 °C for 10 s, 65 °C for 15 s, and 72 °C for 30 s. A melting curve analysis was conducted, and the expression of RBP4 and GLUT4 was normalized against that of *GAPDH*. The results are displayed as fold-change compared with the control group. The relative concentration (fold-change) of RBP4 and GLUT4 mRNA was computed using the 2^−∆∆CT^ method [35].

### 2.10. Statistical Analysis

SPSS Statistics version 26 (IBM, SPSS Inc., Chicago, IL, USA) was used to conduct statistical analyses. Differences between groups were assessed by a one-way analysis of variance followed by Duncan’s post hoc test. Mean ± standard error was used to express statistical assessments of the data. *p* < 0.05 was considered statistically significant.

## 3. Results

### 3.1. High-Performance Liquid Chromatography

**Figure 1:**
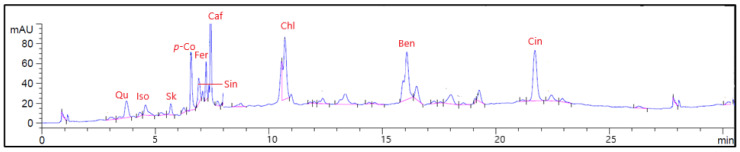
HPLC-DAD of methanolic extract of GCBE. Qu: Quinic acid, Iso: Isocitric acid, Sk: Shikimic acid, *p*-Co: *p*-Coumaric acid, Sin: Sinapic acid, Fer: Ferulic acid, Caf: Caffeic acid, Chl; Chlorogenic acid, Ben: 3,4-Dihydroxybenzoic acid, Cin: Cinnamic acid.

The identification and quantitation of phenolic compounds of GCBE were performed using high-performance liquid chromatography (HPLC-DAD) [36,37]. The HPLC-DAD chromatogram for the organic and phenolic compounds present in GCBE is shown in Figure 1. The major identified phenolic compounds in μg/mg were chlorogenic acid (184.07), followed by cinnamic acid (136.03), caffeic acid (56.16), and *p*-coumaric acid (25.23) as listed in Table 1.

### 3.2. Effects of GCBE on Organ and Body Weight

**Figure 2:**
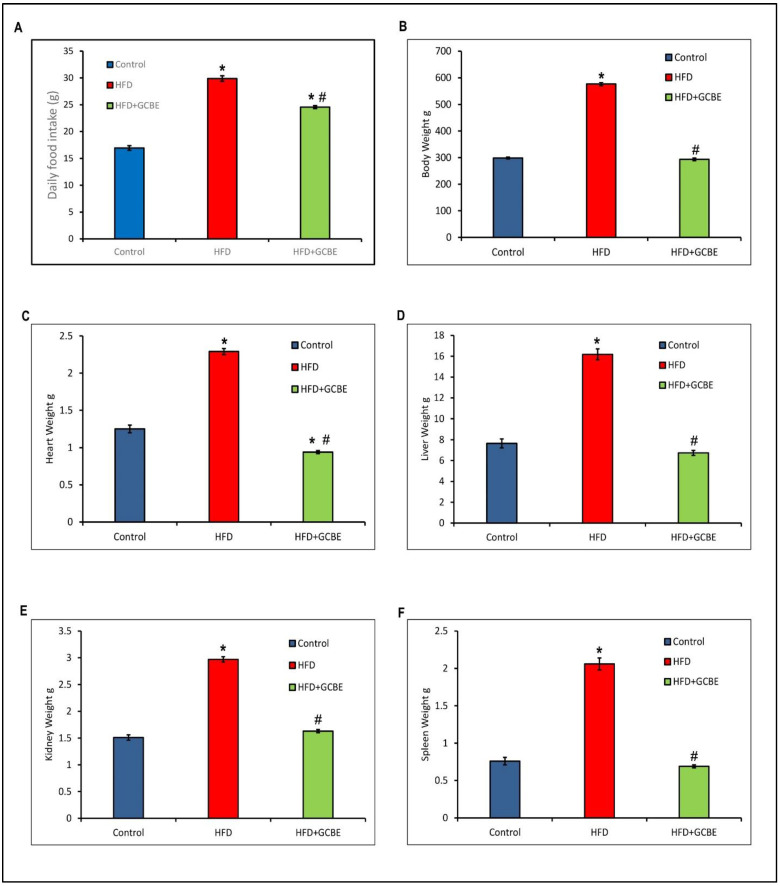
GCBE ameliorative effect on (**A**) Daily food intake, (**B**) Body weight, (**C**) Heart weight, (**D**) Liver weight, (**E**) Kidney weight, and (**F**) Spleen weight in HFD induced obese rats for eight weeks. Data are expressed as the mean ± SE, n = 8. * *p* < 0.05, compared with control; and # *p* < 0.05, compared with HFD.

Figure 2A revealed that the HFD group manifested a significant (*p* < 0.05) increase in daily food intake compared to the control group. For example, rats fed HFD with oral administration of GCBE showed a significant (*p* < 0.05) decline in daily food intake compared to the HFD group. There was a significant (*p* < 0.05) increase in the daily food intake of the HFD + GCBE rats compared with control rats.

Body weights of rats fed a control diet, an HFD, and an HFD with GCBE were compared (Figure 2B). The bodyweight of the HFD group was greater than that of the control group. Compared with the HFD group, the bodyweight of the HFD + GCBE group was considerably (*p* < 0.05) lower. In contrast, the bodyweight of the HFD + GCBE rats and control rats did not differ significantly.

A notable effect of GCBE on the weight of the heart, liver, kidney, and spleen was evaluated against the HFD group (Figure 2C–F, respectively)**.** Rat organ weights were considerably higher in the HFD group than in the control group (*p* < 0.05). Nonetheless, the heart, liver, kidney, and spleen weights in the HFD + GCBE group were significantly decreased compared with the HFD group (*p* < 0.05), but there were no substantial differences between the liver, kidney, and spleen weights in the HFD + GCBE group and the control group. In contrast, the heart weight in the HFD + GCBE group displayed a marked (*p* < 0.05) decrease compared to the control group. The individual data of each group was presented in Table S2. Also, photos of rats showing differences in visceral fat accumulation were displayed in Figure S1.

### 3.3. Effects of GCBE on Serum Metabolite Profile

GCBE alleviated chronic HFD-induced insulin resistance, hyperlipidemia, and inflammation, and an ameliorative effect of GCBE was observed on the metabolic parameters in the serum (Table 2). Biochemical analysis of serum TC, TG, LDL-C, VLDL-C, glucose and insulin levels, and HOMA-IR in the control group were markedly lower than in the HFD group (all *p* < 0.05). In contrast, adiponectin levels in the control group were significantly improved. Furthermore, there was no substantial change in HDL-C levels between the HFD and control groups.

According to the lipid analysis, HFD + GCBE treatment resulted in a marked decrease in TC, TAG, LDL-C, and VLDL-C levels (all, *p* < 0.05) compared with the HFD group. Rats administered green coffee exhibited a significant decline in TAG and LDL-C levels compared with control rats. Nevertheless, no differences were observed between TC and VLDL-C levels between control rats and GCBE-treated rats. Compared with the HFD and control groups, HDL-C levels in the HFD + GCBE group were significantly elevated.

The fasting serum glucose level in the HFD + GCBE group was significantly decreased (*p* < 0.05) compared with the HFD group. GCBE-treated rats had markedly lower serum glucose levels compared with the control rats. The concentration of serum insulin in the HFD + GCBE group was significantly (*p* < 0.05) lower than in the HFD group. Moreover, a significant decrease in insulin levels was apparent compared with the control. The HFD group exhibited insulin resistance as evidenced by a substantial increase in HOMA-IR; however, GCBE treatment caused a marked reduction in HOMA-IR compared with the HFD group. Also, there was a significant decrease in HOMA-IR of GCBE-treated animals compared with the controls (*p* < 0.05).

In addition to ameliorating insulin resistance and lipid abnormalities, the administration of GCBE with HFD significantly reversed serum adiponectin levels (anti-inflammatory adipokine) compared to the HFD group. There was also a notable (*p* < 0.05) enhancement in adiponectin level resulting from the administration of GCBE + HFD compared with control rats. 

### 3.4. The Effects of GCBE on the Morphology of Epididymal Adipose Tissue

**Figure 3:**
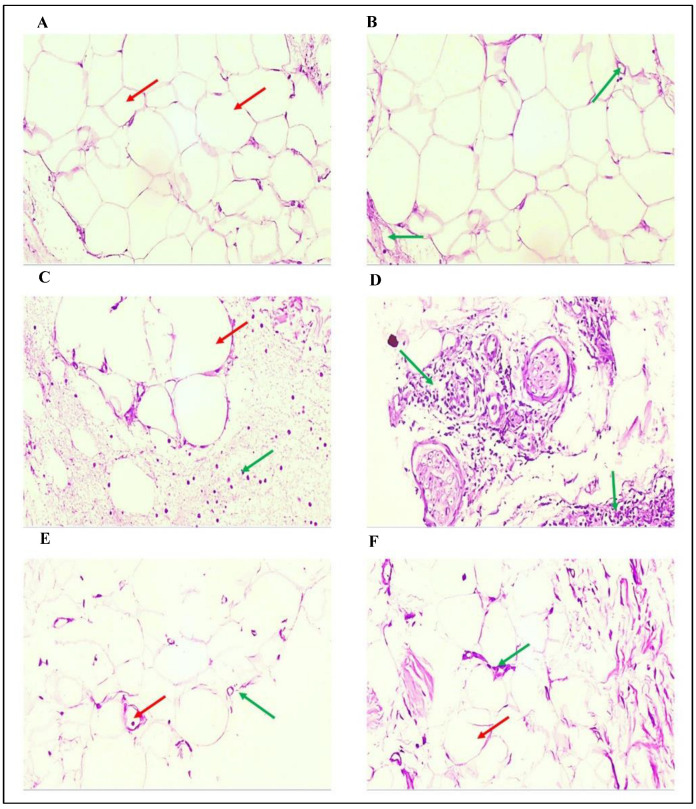
Histological characteristics of epididymal adipose tissue of H&E-stained adipose tissue sections were viewed under a microscope (×100). (**A**,**B**) Sections from the control group showed the average size of adipocytes separated by fine fibrous septa [red arrow] in (**A**) and containing small-sized blood vessels without congestion or inflammation [green arrow] in (**B**). (**C**,**D**) Sections from HFD induced obese rats (HFD group) showed a large size of adipocytes [red arrow] surrounded by inflammatory cells, mainly histocytes (macrophages) in (**C**). Also, (**D**) revealed infiltration with heave inflammatory cells, mainly macrophages [green arrow]. (**E**,**F**) Sections from HFD-induced obese rats after administration of GCBE (HFD + GCBE) displayed a reduction in the size of adipose tissue volume with average-sized adipocytes [green arrow] with normal-sized blood vessels (no congestion) [red arrow] in (**E**). Then (**F**) exhibited average-sized adipocytes [red arrow] separated with thin fibrous septa without inflammation containing normal-sized blood vessels (no congestion) [green arrow] (H&E × 100).

**Figure 4:**
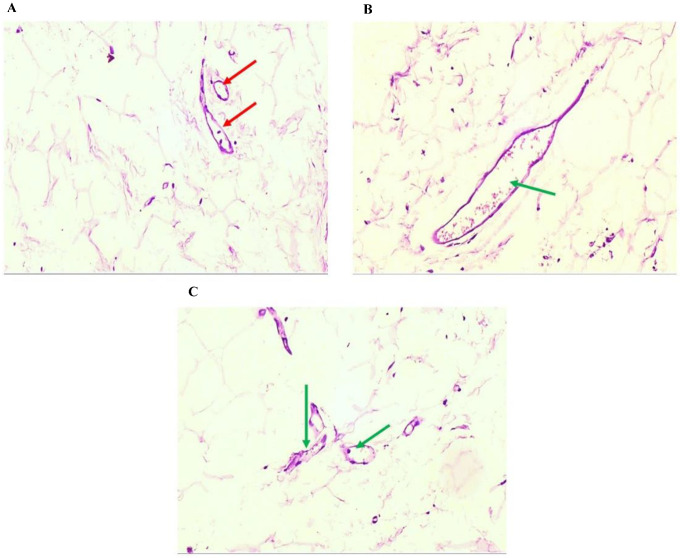
Microscopical images of epididymal adipose tissue of H&E stained sections of adipose tissue were viewed under a microscope (×400). (**A**) Higher magnification of normal adipose tissue (control group) showed average-sized blood vessels without congestion [red arrows]. (**B**) Higher magnification of obese adipose tissue (HFD group) showed large dilated congested blood vessels [green arrow]. (**C**) Higher magnification of adipose tissue (HFD + GCBE) showed average-sized blood vessels with no congestion [green arrows] (H&E × 400).

The histological changes in adipose tissue because of GCBE administration with HFD were examined to confirm the above findings. Average-sized adipocytes separated by fine fibrous septa containing small-sized blood vessels without congestion or inflammation were observed in control cells (Figure 3A,B and Figure 4A). In contrast, the HFD group exhibited large-sized adipocytes surrounded by inflammatory cells, mainly histocytes, and large dilated congested blood vessels (Figure 3C,D and Figure 4B). In the HFD + GCBE group, a decrease in the size of the adipose tissue volume with average-sized adipocytes separated with thin fibrous septa, no inflammation, and containing normal-sized blood vessels with no congestion were observed (Figure 3E,F and Figure 4C). The histopathological findings were consistent with the biochemical data (Table 2), indicating that GCBE administration has a considerable ameliorative effect on HFD-induced obesity, hyperlipidemia, insulin resistance, and inflammation.

### 3.5. Effects of GCBE on mRNA Expression in Adipose Tissue Genes

**Figure 5:**
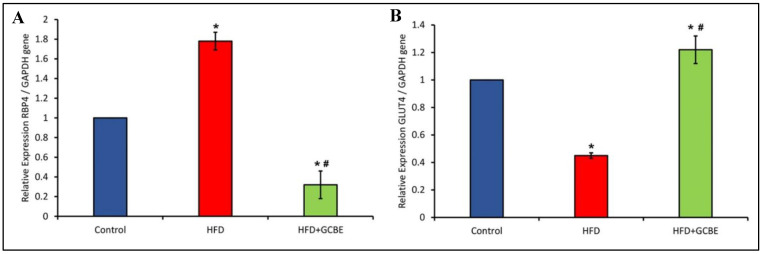
The ameliorative effect of GCBE administration on (**A**) Expression of RBP4 gene showed a reduction of the elevated mRNA expression level of RBP4 in epidydimal WAT of obesity-induced rats. (**B**) Expression of the GLUT4 gene showed enhancement of the obesity-related decrease in GLUT4 expression in epidydimal adipose tissue of obesity-induced rats. The expression level of RBP4 and GLUT4 mRNA was assessed by RT-PCR. Data are shown as the mean ± SE, n = 8. * *p* < 0.05, compared with control; and # *p* < 0.05, compared with HFD.

The mRNA expression levels in the epididymal adipose tissues were measured using quantitative real-time PCR. We determine the GCBE effect on the expression of the RBP4 and GLUT4 genes (Figure 5A,B). As shown in Figure 5A, the consumption of an HFD significantly increased (*p* < 0.05) RBP4 mRNA levels in epidydimal adipose tissue; however, this elevation was significantly (*p* < 0.05) ameliorated in the GCBE + HFD group compared with the HFD group. In contrast, the HFD + GCBE group exhibited considerably (*p* < 0.05) lower levels of RBP4 expression in the epidydimal White adipose tissue (WAT) compared with the control group.

The GLUT4 expression in adipose tissue of induced obese rats was markedly lower compared with the HFD + GCBE group and control rats (Figure 5B). GCBE administration with HFD was associated with a decline compared with the HFD rats. Furthermore, The HFD + GCBE group had considerably higher levels of GLUT4 expression in the epidydimal WAT compared with the control group.

Taken together, these observations indicate that administering a dose of 200 mg GCBE /kg significantly reduces RBP4 expression and enhances GLUT4 expression in epidydimal adipose tissue of HFD-induced obese rats.

## 4. Discussion

Studies indicate that polyphenols containing plants like green coffee may be used to treat various health problems, including metabolic syndrome. The beneficial health effects of GCBE are attributed to the antioxidant activity of numerous polyphenol components and a significant contribution of CGA [11]. This research was done to elucidate the effect of 200 mg of GCBE/kg BW for eight weeks on obesity, the alleviation of insulin resistance, and inflammation resulting from obesity. Our data demonstrated that the HFD group manifested a significant (*p* < 0.05) increase in daily food intake compared to the control group. For example, rats fed HFD with oral administration of GCBE showed a significant (*p* < 0.05) decline in daily food intake compared to the HFD group. In addition, there was a significant (*p* < 0.05) increase in the daily food intake of the HFD + GCBE rats compared with control rats. This result is supported by a previous study [38]. Contrary to our results, Choi et al. [16] reported that six weeks of GCBE administration with HFD did not affect the food intake in HFD-induced obese mice.

Consistent with our hypothesis, when compared to the control group, the bodyweight of the HFD group was increased. Compared with the HFD group, the bodyweight of the HFD + GCBE group was significantly lower. Consistent with our results, the bodyweight of animals nourished with HFD for four weeks was significantly increased compared with control rats maintained on a normal diet. In addition, the administration of green coffee induced a remarkable reduction in body weight compared with the obese group when administrated to obese rats for six weeks [39]. Another study of obese mice treated with an HFD + 100 or 200 mg/kg GCBE for six weeks indicated reduced body weight compared with the HFD group [16]. However, in a mouse model, 5% (*w*/*w*) GCBE + HFD did not lose weight after 12 weeks [40]. Our study resulted in a remarkable finding that there were no notable changes between the bodyweight of HFD with GCBE and control rats. This indicates that GCBE has a remarkable restorative effect on obese rats. The mechanisms postulated for the GCBE effects on weight loss were explained by Ríos-Hoyo and Gutiérrez-Salmeán [19]. These include a lipolytic action on adipocytes, a reduction in pancreatic lipase activity, and inhibition of fatty acid synthase, hydroxymethyl-glutaryl-coenzyme A reductase (HMG-COA Reductase), and acyl-coenzyme A: cholesterol acyltransferase (ACAT), an increase in *β*-oxidation, and elevation of PPAR-*α* expression in the liver. Furthermore, Song et al. [17] first observed that GCBE intake helps control appetite, leading to weight loss. Green coffee bean extract was found to restrict bodyweight gain more effectively than chlorogenic acid or caffeine, which was ascribed to the synergistic effect of caffeine and chlorogenic acid in CGBE [41].

In the present study, rat organ weight was markedly higher in the HFD group compared with the control group. Nonetheless, a dramatic reduction was observed regarding heart, liver, kidney, and spleen weight in the HFD with GCBE group compared with the HFD group. Based on our findings, the liver weight of rats in the HFD group was higher than that of a normal diet; however, the HFD with the GCBE group exhibited reduced liver weight. The group treated with GCBE at 200 mg/kg showed a marked decline in liver weight compared with the HFD group after six weeks [16]. Likewise, GCBE had a potent restorative action indicating no considerable variation between the weight of the liver, kidney, and spleen in the HFD + GCBE versus the control group. In addition, the heart weight in the HFD + GCBE group displayed a marked decrease compared with the control group.

According to our study, the serum TC, TAG, LDL-C, and VLDL-C levels in the control group were considerably less compared with the HFD group, and there was no significant difference between HDL-C levels in the HFD group and the control group. The administration of GCBE with HFD decreased TC, TAG, LDL-C, and VLDL-C levels markedly compared with HFD-fed rats. In addition, there was a significant elevation in HDL-C levels compared with the HFD and control rats. Some animal studies have indicated positive effects. For example, TC, TG, and LDL-C levels in the control group were considerably lower compared with the HFD group. Treatment with 200 mg GCBE /kg and HFD for 6 weeks reduced TC, TAG, and LDL-cholesterol levels while increasing HDL-cholesterol levels compared with an HFD group [16]. Another study revealed that 10 mg/kg BW/day of GCBE for 13 days was an effective dose for improving the lipid profile of HFD exacerbated obese rats [42]. Consistent with our study, green coffee administrated rats exhibited a considerable decline in TAG and LDL-C levels compared with control rats. There was a marked increase in HDL-C levels compared with the control group. Furthermore, no variations were observed in TC and VLDL-C levels between control rats and green coffee-treated rats. This indicates that the remedial activity of GCBE is robust.

We found that GCBE reduced fasting serum glucose levels and limited its increase compared with control rats. This is consistent with a study of the effects of decaffeinated GCBE on mice provided an HFD diet containing 1%, 3%, or 9% GCBE. After 11 weeks, the group receiving 3% GCBE with an HFD diet presented a marked reduction in FSG compared with the HFD group [17]. Another study evaluated the effects of light and dark roasted coffee on diabetic rats. Green coffee was the most effective at lowering glucose levels. In another animal study, fasting glucose Levels in CGA-treated mice were significantly lower than in HFD-fed mice [23].

Moreover, 100 mg/kg GCBE + HFD resulted in a profound reduction in FSG after 6 weeks compared with an HFD group [16]. Our study found that GCBE-treated rats had significantly higher serum glucose levels than control rats. This suggests that GCBE has strong restorative effects on obese rats. In addition, CGBE lowers FSG by activating AMP-activated protein kinase (AMPK). AMPK is a protein kinase essential for cellular and systemic energy balance [43]. When AMPK is activated, GLUT4 translocates to the plasma membrane, resulting in increased glucose transport into cells and peripheral glucose elimination [44]. CGA in GCBE also suppresses glucose-6-phosphatase (G6Pase), which functions in blood glucose regulation, resulting in limited glucose synthesis through gluconeogenesis and glycogenolysis [42].

In the current investigation, it was also discerned that serum insulin level in the control group was substantially less than in the HFD, and the serum insulin level in the HFD + GCBE group exposed a statistically significant decline compared with the HFD group. It was also pointed out by Choi et al. [16] that both body weight and epididymal adipose weight were directly correlated with insulin levels. The HFD group’s plasma insulin level was significantly more significant than the control group. However, chlorogenic acid supplementation dramatically reduced this hormone level. Another study revealed that CGA alleviated hyperinsulinemia in mice fed with an HFD [45]. GCBE exerts a potent ameliorative effect that was evident by a marked decrease in insulin levels of GCBE-treated animals relative to controls.

Interestingly, our findings indicate that GCBE administration exerts a marked reduction in HOMA-IR compared with the HFD group. Thus, GCBE administration enhanced insulin sensitivity in obese rats, which was confirmed by alleviating obesity-related hyperinsulinemia and HOMA-IR decline. Several studies have supported these conclusions. For example, in one study, mice given DGCBE at 80 mg/kg for 14 weeks showed improved HFD-induced insulin resistance [20]. In contrast, a 12-week study using the HFD-induced Mets mouse model did not demonstrate increased insulin resistance in mice maintained on an HFD diet containing 05% (*w*/*w*) GCBE [46]. Also, a marked decrease in HOMA-IR following GCBE administration compared to controls, indicating that GCBE has a remarkable remedial activity on obese rats. In addition, the chronic exposure of adipocytes to modest oxidative stress lowers glucose metabolism and causes insulin resistance. Consequently, oxidative stress directly and negatively impacts insulin’s function in glucose transport.

Antioxidant consumption can alleviate glucose tolerance and insulin levels. Song et al. [17] indicated that GCBE has a progressive impact on insulin resistance through a decline in phosphorylation of c-Jun N-terminal kinase, which results in the activation of insulin receptor substrate-1 (IRS1), leading to GLUT4 translocation to the membrane of adipocytes and increasing insulin sensitivity.

An important finding from our study was that adiponectin levels in the control group were considerably elevated compared with the HFD group. The administration of GCBE with HFD ameliorated adiponectin levels considerably compared with the HFD group. Consistently, HFD decreases plasma adiponectin levels. Nevertheless, chlorogenic acid considerably elevated the plasma adiponectin concentration following the 8-week trial compared with the HFD group [21]. The present study provided an important result of a notable enhancement in adiponectin level resulting from the administration of GCBE with HFD compared with control rats. This indicates that GCBE has a potent restorative effect. Adiponectin, a well-known adipokine, is vital for metabolic health. Therefore, it is suggested to have antiatherogenic, anti-inflammatory, and anti-diabetic properties. Low adiponectin levels in the serum are related to central obesity, insulin resistance, type 2 diabetes, and metabolic syndrome [47]. Evidence that adiponectin increases glucose uptake and fatty acid oxidation in myocytes while lowering hepatic glucose synthesis supports its importance in improving insulin sensitivity.

Moreover, it causes catabolism of VLDL in skeletal muscle and results in a decrease in plasma triglycerides [48]. Adiponectin exerts its effects by activating the AMPK and the peroxisome proliferator-activated receptor (PPAR) [49]. In addition, adiponectin may regulate appetite through the melanocortin pathway [50].

The histological observation of adipose tissue revealed that the HFD + GCBE group had smaller-sized adipocytes with no inflammation containing normal-sized blood vessels with no congestion compared with the HFD group, which exhibited large-sized adipocytes surrounded by inflammatory cells and large dilated congested blood vessels. This observation is consistent with Choi et al. [16], who mentioned a significant reduction in adipocyte hypertrophy compared with animals fed an HFD alone. This decrease in hypertrophy restricts the stimulation of lipolysis, reduces macrophage infiltration, decreases the expression of inflammatory cytokines in adipose tissue, increases pro-inflammatory cytokines, and enhances insulin resistance in HFD-stimulated obese rats [51].

According to recent studies, various members of the lipocalin family contribute to whole-body metabolism. Serum retinol-binding protein, which is a 21-kD protein produced in the liver and adipose tissue, acts primarily as a transporter of retinol or vitamin A. RBP4 is an adipocyte-secreted hormone that is enhanced in insulin resistance associated with obesity and also induces insulin resistance [52]. This study indicated that the immune system, specifically antigen-presenting cells, such as dendritic cells, macrophages, and CD4 T cells, were mediators of the inflammatory response triggered by RBP4 in adipose tissue [51]. Visceral fat expresses RBP4 at a higher level than subcutaneous fat, suggesting that RBP4 may be a marker for intra-abdominal fat accumulation, which is thought to exacerbate the underlying pathogenesis of metabolic syndrome [53]. Two possible factors explain why high circulating RBP4 results in elevated blood glucose. The first is its ability to suppress skeletal muscle insulin signaling by reducing phosphoinositide-3-kinase (PI-3-kinase) activity and subsequent phosphorylation of the insulin receptor substrate-1 (IRS-1), which are indispensable constituents of the insulin signaling pathway. The second factor is elevated hepatic glucose output by enhancing the PEPCK enzyme expression, which leads to increased hepatic glucose output, which elevates blood glucose [54]. The influence of GCBE administration with HFD on RBP4 expression in adipose tissue has not been investigated. Our study yielded a significant new finding that the consumption of HFD elevated RBP4 mRNA expression in the epidydimal adipose tissue; however, this elevation was significantly improved by administration of GCBE with HFD compared with the HFD group. However, the HFD + GCBE group had considerably decreased levels of RBP4 gene expression in the epidydimal WAT compared with the control group. Our findings support previous studies showing that GCBE exerts anti-insulin resistance and anti-inflammatory effects. We hypothesize that this effect results from the ability of GCBE to decrease the weight of visceral fat, as revealed in Lukitasari et al. [21]. When comparing the chlorogenic acid group to the HFD group, the weight of the epididymal and perirenal adipose tissues was considerably lower in the chlorogenic acid group by 46% and 42%, respectively. Another study indicated that HFD with GCBE treatment of rats lowered perirenal, retroperitoneal, epididymal, and total WAT weight compared with HFD-treated rats [16]. The histological analysis confirmed that GCBE ameliorates and restores adipose tissue to normal, reducing the production of pro-inflammatory cytokines, such as RBP4, and increasing the production of anti-inflammatory cytokines, including adiponectin.

We supposed that administration of GCBE would be profitable for enhancing GLUT4 expression in adipose tissue of HFD-stimulated obese rats, based on Meng et al. [55], which revealed that GCBE stimulates the GLUT4-related genes expression and proteins implicated in GLUT4 translocation at epididymal adipose tissues, after which the function of insulin receptors in tissues is improved. Our data investigated the effect of GCBE on GLUT4 expression in adipose tissue for the first time. In line with our premise, the mRNA expression of GLUT4 in adipose tissue of HFD-provoked obese rats was observably lower than in control rats. Markedly, GCBE administration with HFD upraised this decline compared with the HFD group. The ameliorative activity of GCBE is powerful, as illustrated by the HFD with the GCBE group that had notably higher levels of Glut4 gene expression in the epidydimal WAT than the control group. The function of GLUT4, the insulin-sensible glucose transporter, is glucose uptake. It is found predominantly in skeletal muscle and adipose tissue, and the rate at which it is expressed is a significant determinant of muscle glucose uptake capacity. In addition, GLUT4 overexpression in muscle promotes glucose absorption and lowers insulin resistance. In addition, RBP4 and interleukin-6 (IL-6) are inflammatory cytokines produced by adipose tissue linked to a decrease in GLUT4 expression [56]. This explains the negative correlation between the RBP4 and GLUT4 in this investigation. When the WAT becomes inflamed, c-Jun N-terminal kinase (JNK) is phosphorylated, and p-JNK phosphorylates the serine residue of insulin receptor substrate-1, deactivating it. Serine phosphorylation prevents GLUT4 translocation, which diminishes insulin sensitivity. GCBE reduced JNK activation and increased GLUT4 translocation to counteract insulin resistance caused by a high-fat diet [56].

## 5. Conclusions

Our findings implied that GCBE could improve body weight, organ weight, lipid profile, insulin resistance, HOMA-IR, adiponectin levels in serum, RBP4, and GLUT4 expression in adipose tissue of obese rats persuaded by HFD. Accordingly, green coffee extract administration could, in a feasible manner, be an efficient option for managing obesity and some concerning disorders such as dyslipidemia, insulin resistance, and inflammation. Future preclinical and clinical investigations on GCBE should demonstrate its efficacy in treating obesity disorders. In addition, it is essential to formulate GCBE in different pharmaceutical dosage forms to maximize their practical incorporation into the obesity treatment regimens.

## Figures and Tables

**Table 1:** Chemical composition of GCBE phenolic and organic acid components by HPLC-DAD.

No	Compound	Concentration (μg/mg)
1	Quinic acid	3.07
2	Gallic acid	ND
3	Isocitric acid	2.27
4	Shikimic acid	1.98
5	Protocatechuic acid	ND
6	Gentisic acid	ND
7	*p*-coumaric acid	25.23
8	Caffeic acid	56.16
9	Ellagic acid	ND
10	Vanillic acid	ND
11	Sinapic acid	5.89
12	Ferulic acid	6.94
13	Syringic acid	ND
14	Chlorogenic acid	184.07
15	3,4-Dihydroxybenzoic acid	8.31
16	Rosmarinic acid	ND
17	Cinnamic acid	136.03

**Table 2:** Levels of metabolic parameters of serum in obese rats fed an HFD after administration of GCBE.

Parameters	Experimental Groups		
	Control	HFD	HFD + GCBE
TC (mg/dL)	76.57 ± 2.21 ^b^	178.07 ± 2.40 ^a^	80.63 ± 2.97 ^b^
TAG (mg/dL)	81.95 ± 3.94 ^b^	128.94 ± 2.78 ^a^	68.79 ± 3.81 ^c^
HDL-C (mg/dL)	26.36 ± 1.91 ^b^	30.51 ± 0.93 ^b^	42.82 ± 1.44 ^a^
LDL-C (mg/dL)	33.82 ± 1.21 ^b^	121.77 ± 2.93 ^a^	23.52 ± 3.15 ^c^
VLDL-C (mg/dL)	16.39 ± 0.79 ^b^	25.79 ± 0.56 ^a^	14.26 ± 0.99 ^b^
FSG (mg/dL)	94.23 ± 3.92 ^b^	138.59 ± 1.58 ^a^	76.02 ± 3.63 ^c^
Insulin (uIU/mL)	20.46 ± 0.35 ^b^	43.61 ± 0.52 ^a^	17.76 ± 0.60 ^c^
HOMA-IR	4.45 ± 0.24 ^b^	15.02 ± 0.27 ^a^	3.30 ± 0.07 ^c^
Adiponectin (ng/mL)	132.50 ± 1.73 ^b^	80.25 ± 1.31 ^c^	139.55 ± 2.62 ^a^

The above parameters were examined with the serum of control, HFD, and high-fat diet with green coffee bean extract (HFD + GCBE) groups. TC: total cholesterol, TAG: triacylglycerol, HDL-C: high-density lipoprotein-cholesterol, LDL-C: low-density lipoprotein-cholesterol, VLDL-C: very-low-density lipoprotein-cholesterol, FSG: fasting serum glucose, and HOMA-IR: homeostasis model assessment-insulin resistance. All values are expressed as the mean ± SE, n = 8. Statistically significant difference *p* < 0.05 was remarked by superscript letters in the same raw. (a) is significantly different than (b), and (b) is significantly different than (c).

## Data Availability

Data is contained within the article and supplementary materials.

## Supplementary Materials

The following supporting information can be downloaded at: https://www.mdpi.com/article/10.3390/life12050693/s1, Table S1. The sequences of the primers, Table S2. The individual data of each group shows GCBE effect on Daily food intake, Bodyweight, Heart weight, Liver weight, Kidney weight, and Spleen weight, Figure S1. Photos of rats showing differences in visceral fat accumulation.

## Abbreviations

ACAT	acyl-CoA cholesterol acyltransferase
AMPK	Adenosine monophosphate-activated protein kinase
ANOVA	Analysis of variance
CD4	Cluster of differentiation 4
CESR	Center of Excellence for Scientific Research
CGA	Chlorogenic acids
DNA	Deoxyribonucleic Acid
ELISA	Enzyme-linked immunosorbent assay
FSG	Fasting serum glucose
GAPDH	Glyceraldehyde-3-Phosphate Dehydrogenase
GCBE	Green coffee bean extract
GLUT4	Glucose transporter isoform 4
G6Pase	Glucose-6-phosphatase
HMG-COA Reductase	Hydroxy methyl-glutaryl-coenzyme A reductase
HOMA-IR	homeostasis model assessment-insulin resistance
HFD	High fat diet
IL-6	Interleukin-6
IL-10	Interleukin-10
IRS1	Insulin receptor substrate-1
JNK	c-Jun N-terminal kinase
Mets	The metabolic syndrome
mg/dL	Milligrams per deciliter
mRNA	Messenger Ribonucleic Acid
ng/mL	Nanograms per milliliter
PEPCK	Phosphoenolpyruvate carboxykinase
PI-3-kinase	Phosphoinositide-3-kinase
p-JNK	Phosphorylated c-Jun N-terminal kinase
PPAR-α	peroxisome proliferator-activated receptor- Alpha
qPCR	Quantitative polymerase chain reaction
RBP4	Retinol binding protein 4
SPSS	Statistical Package for the Social Sciences
T2DM	Type 2 diabetes mellitus
uIU/mL	Micro International Units per milliliter
WAT	White adipose tissue
