# Supplementary material for: Green Coffee Bean Extract Normalize Obesity-Induced Alterations of Metabolic Parameters in Rats by Upregulating Adiponectin and GLUT4 Levels and Reducing RBP-4 and HOMA-IR
Source: Life (Basel). 2022 May 6;12(5):693. doi: 10.3390/life12050693 (PMC9144088; doi:10.3390/life12050693)
Supplement: Supplementary file 1 [file life-12-00693-s001.zip › life-1652532-supplementary.pdf]

Green Coffee Bean Extract Normalize Obesity-Induced Alterations of Metabolic Parameters in Rats by Upregulating Adiponectin and GLUT4 Levels and Reducing RBP-4 and HOMA-IR

Esraa M. Seliem [1], Mohamed E. Azab [1], Randa S. Ismail [1], Abeer A. Nafeaa [1], Badriyah S. Alotaibi[2,*], Walaa A. Negm [3,*]

[1] Department of Physiology, Faculty of Veterinary Medicine, Benha University, Benha 13512, Egypt.
[2] Department of Pharmaceutical Sciences, College of Pharmacy, Princess Nourah bint Abdulrahman University, P.O. Box 84428, Riyadh 11671, Saudi Arabia; bsalotaibi@pnu.edu.eg (B.A.)
[3] Department of Pharmacognosy, Faculty of Pharmacy, Tanta University, Tanta 31111, Egypt
* Correspondence: bsalotaibi@pnu.edu.eg (B.A.); walaa.negm@pharm.tanta.edu.eg (W.A.N.).

Table S1. The primers sequences used for qPCR

Gene	Forward primer (5'→ 3')	Reversed primer (3'→ 5')
**RBP4**	TGATCGTCCACAACGGTTAC	GAGCTGAAGACTGAGAGCTAATC
**GLUT4**	CCATAGGAGCTGGTGTGGTCAATAC	TCGCCCAGCTCGCTCTACTAAG
**GAPDH**	GGTGTGAACCATGAGAAGTATGA	GAGTCCTTCCACGATACCAAAG

Table S2. The individual data of each group shows GCBE effect on Daily food intake, Body weight, Heart weight, Liver weight, Kidney weight, and Spleen weight

Groups	Daily food intake	Body weight	Liver weight	Spleen weight	Kidney weight	Heart weight
1.00	16.96	310.00	6.00	0.65	1.50	1.19
1.00	16.98	305.00	8.50	0.62	1.80	1.21
1.00	16.91	305.00	6.21	0.65	1.64	1.18
1.00	17.01	303.00	8.80	0.84	1.34	1.31
1.00	16.86	300.00	8.84	0.72	1.43	1.01
1.00	16.51	290.00	6.50	0.70	1.34	1.42
1.00	17.37	280.00	7.81	0.89	1.50	1.40
1.00	16.92	295.00	8.50	0.98	1.50	1.29
2.00	29.36	575.00	15.50	1.92	2.86	2.26
2.00	29.46	580.00	14.90	1.95	3.13	2.16
2.00	29.88	550.00	17.20	1.81	3.23	2.30
2.00	30.50	570.00	15.71	1.85	2.81	2.44
2.00	30.48	600.00	14.91	2.19	2.95	2.43
2.00	30.40	590.00	14.70	2.10	2.85	2.17
2.00	29.22	595.00	18.00	2.50	2.90	2.19
2.00	29.96	555.00	18.48	2.16	3.00	2.37
3.00	24.97	285.00	6.33	0.66	1.57	0.92
3.00	24.56	280.00	7.00	0.67	1.50	0.93
3.00	24.15	292.00	7.10	0.69	1.51	0.91
3.00	24.28	270.00	8.01	0.67	1.71	0.96
3.00	25.02	310.00	7.10	0.69	1.72	0.89
3.00	23.97	305.00	6.00	0.62	1.66	0.86
3.00	24.59	305.00	6.15	0.70	1.70	1.00
3.00	24.45	300.00	6.25	0.81	1.66	1.01

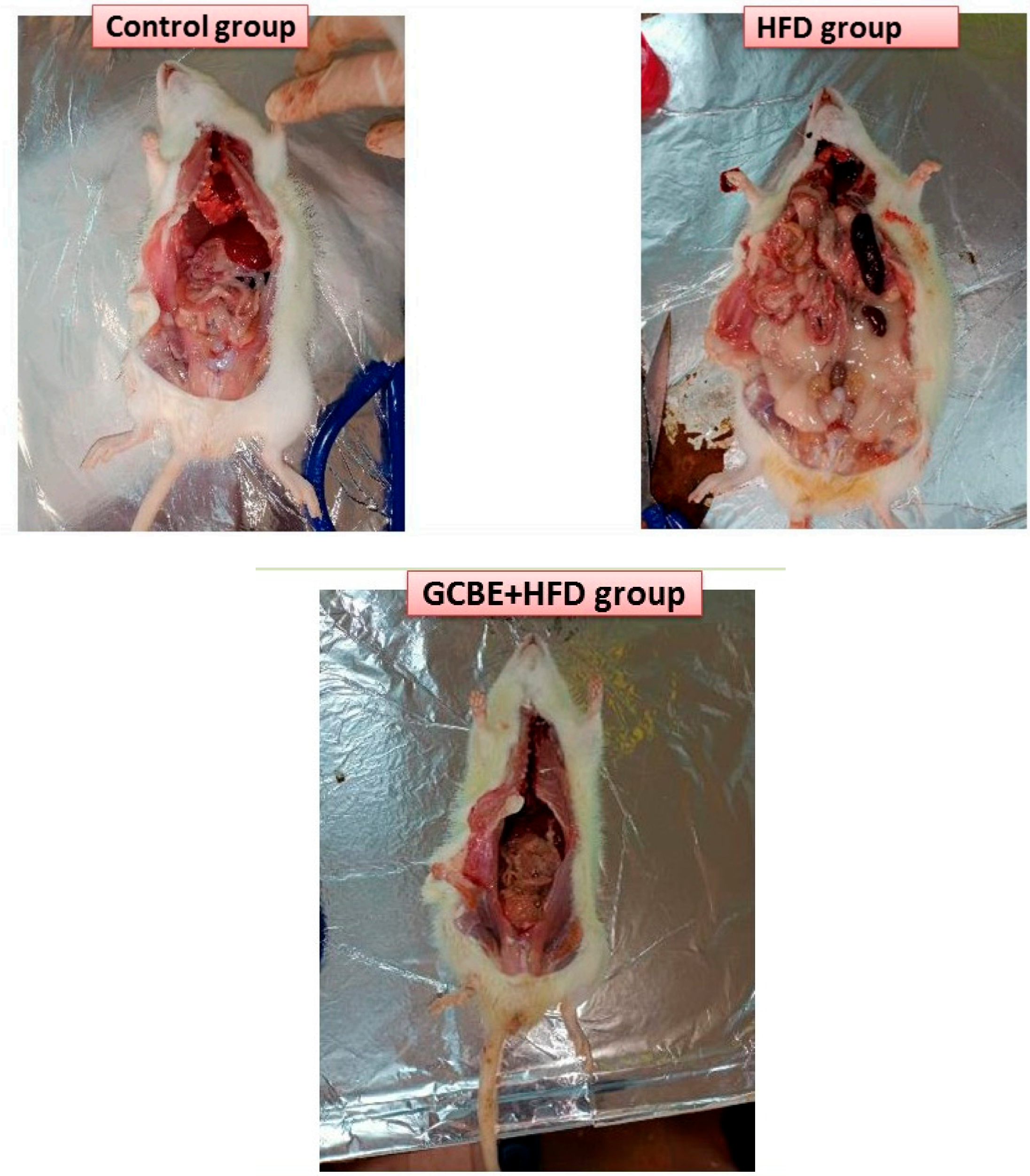

Figure S1. Photos of rats showing differences in visceral fat accumulation

There is an observable difference between the body and organs weight between the control group and the high-fat diet (HFD) group. The rats in the HFD group have a remarkable increase in visceral fat accumulation. This confirmed that the observed differences in body weight or particular organs because of obesity and not reflect growth speed.
